# 360°-Based Cognitive-Motor Training System for Older Adults With Cognitive Impairment: User-Centered Design and Evaluation Study

**DOI:** 10.2196/68032

**Published:** 2026-03-13

**Authors:** Francesco Bigotto, Jonathan Panigada, Francesca Bruni, Valentina Mancuso, Silvia Cavedoni, Marco Stramba-Badiale, Karine Marie Goulene, Elisa Pedroli, Silvia Serino

**Affiliations:** 1 Older People Rehabilitation and Cerebrovascular Medicine Research Laboratory IRCCS Istituto Auxologico Italiano Milano, Lombardia Italy; 2 Department of Theoretical and Applied Sciences eCampus University Novedrate Italy; 3 Department of Medicine, Neurology and Rehabilitation IRCCS Istituto Auxologico Italiano Milano Italy; 4 Department of Psychology Università degli Studi di Milano-Bicocca Milano Italy

**Keywords:** dual task, user centered, aging, usability, technology, cognitive-motor training

## Abstract

**Background:**

Evidence suggests that older adults can improve dual-task (DT) performance through specific motor-cognitive training programs. Recent technological advancements have facilitated the development of novel rehabilitative DT methodologies. In particular, the DUAL-REHAB project exploits 360° technology to develop ecological, cost-effective DT exercises for clinical and home settings.

**Objective:**

This study aimed to comprehensively understand the DUAL-REHAB project’s end users (older adults with subjective memory complaints [SMC] and mild cognitive impairment [MCI]) and obtain feedback on an initial DUAL-REHAB mobile app prototype using a user-centered design approach.

**Methods:**

The study used a 2-phase procedure. In the first phase (user requirements), we investigated end users’ lifestyles, habits, perceived well-being, technology adoption, and cognitive and cognitive-motor DT training expectations. In the second phase (prototype evaluation), we developed and tested a DUAL-REHAB mobile app prototype to assess its functionality with end users.

**Results:**

Overall, 14 participants were initially recruited for the study. The sample included 7 women with a mean age of 79 (SD 3.79) years and 7 men with a mean age of 82.43 (SD 5.65) years. One female participant was excluded for not meeting the criteria for either group; accordingly, the final sample study included 13 participants, with 8 categorized as MCI and 5 categorized as SMC. Participants reported structured daily routines with high autonomy, although some faced challenges with social connections. Perceived well-being was moderate across physical (mean 2.79, SD 0.97), psychological (mean 3.14, SD 0.86), and cognitive (mean 3.14, SD 0.53) domains. The perceived technology skills were low (mean 2.57, SD 0.94), with low digital literacy (mean 2.21, SD 0.89). Interest in cognitive training was high, with 92.31% (12/13) participants aware of its benefits and demonstrating strong interest (mean 4.15, SD 1.34) and perceived use (mean 4.15, SD 1.28). While only 46.15% (6/13) were aware of the possibility of DT training with technology, 92.31% (12/13) were willing to participate, and 84.61% (11/13) were open to home-based training. The preferred frequency was 2-3 days per week (63.64%, 7/11), with 10-20 minute sessions (8/11, 72.72%). Prototype evaluation revealed specific usability issues related to icon identification, device interaction, and navigation between training days.

**Conclusions:**

We obtained valuable insights into the lifestyles, habits, and technological needs of older adults with MCI and SMC, which will guide DUAL-REHAB training development to align with user needs and capabilities. Our findings emphasize the importance of simplified technological processes with intuitive interfaces. Additionally, structured interaction opportunities during clinical and at-home training could enhance motivation, facilitate timely problem resolution, and address participants’ social needs.

## Introduction

### Background

In recent decades, a growing focus has been on developing and testing effective strategies to mitigate cognitive decline associated with physiological and pathological aging [1]. The global population of older adults (65 years and older) is projected to reach 1.5 billion by 2050 [2], while Alzheimer disease prevalence may exceed 152 million cases [3]. These conditions significantly impact autonomy through various cognitive, behavioral, and motor symptoms [4-6], including deficits in dual-task (DT) performance, namely the ability to perform 2 tasks simultaneously. The DUAL-REHAB project uses 360° technology to address these challenges and develop ecological training environments for cognitive-motor exercises. Our approach is based on a user-centered design (UCD), prioritizing end user involvement throughout the design and development process. Accordingly, this study explores the needs of older adults with mild cognitive impairment (MCI) and subjective memory complaints (SMC) by investigating their lifestyles, habits, perceived well-being, technology adoption, and expectations regarding cognitive and cognitive-motor DT training, while also gathering their feedback through interaction with a prototype mobile app designed for home-based training.

### Cognitive-Motor DT Training: State-of-the-Art and Current Challenges

Deficits in the ability to divide attention, or to perform 2 tasks simultaneously, referred to as DT performance, are early symptoms of dementia that evolve as the disease progresses [7,8]. An interplay exists between motor skills and cognition, with shared brain areas involved in attention, executive functions, and motor control, particularly in the prefrontal and temporal regions [9]. It is suggested that these networks can either process 1 task at a time [10] or that simultaneous engagement in a motor task and a cognitive task could result in interference caused by competing demands for attentional resources [11]. Despite different theoretical accounts, the effect appears to be more pronounced in individuals with cognitive impairments [12], leading to a deterioration in performance. Several studies have highlighted the close relationship between DT impairment alterations and motor dysfunction [13-15], falls [16-18], and a decline in daily activities observed in both normal and pathological aging [13,19]. Additionally, literature indicates that DT impairments could be considered a clinical marker of progression from cognitive impairment to dementia [9,20-22], underscoring the importance of DT skills for both daily functioning and clinical relevance.

There is convincing evidence suggesting that both older adults with Alzheimer disease and MCI can enhance their DT performance, motor skills, and cognitive abilities through specific DT motor-cognitive training programs [23-26]. Moreover, the recent advancements in technological tools have facilitated the development and implementation of novel rehabilitative DT methodologies [27-30]. A recent systematic review [30] indicated that an increasing body of evidence suggested the potential of exploiting technological devices to enhance DT skills across various age-related chronic conditions. Using motion-tracking systems, it is possible to reproduce users’ movements within immersive virtual reality (VR) environments or on computer screens while participants are engaged in cognitive tasks. VR platforms offer realistic and immersive cognitive challenges and multisensory stimulation, enriching the interactive experience [31,32], also in home settings. For instance, Downey et al [33] recently exploited the potential of semi-immersive VR for older adults’ rehabilitation, specifically targeting executive functions. This protocol used both laboratory and simulated real-world conditions to deliver at-home cognitive training focused on DT performance for both adults with normal-hearing and older adults who use hearing aids.

### UCD: A Critical Approach for Effective Technology-Based Interventions

To ensure that technological interventions effectively meet the needs and capabilities of older adults, adopting a UCD approach is essential [34,35]. This methodological framework places end users at the core of the design and development process, systematically incorporating their perspectives, capabilities, and limitations at every stage. The basic premise of UCD is to directly involve end users in shaping the technology-based solutions they will ultimately use, to make them more easily adopted and effective. For instance, Hassandra et al [36] specifically investigated the acceptability, usability, and tolerability of using the VRADA (VR Exercise App for Dementia and Alzheimer’s Patients) system compared to standard care physical and cognitive training. In another study by Nayak et al [37], 4 feasibility themes emerged from qualitative analyses: reasons for participation, difficulties using the technologies, engagement, and motivational values. UCD shares principles with related approaches like human-centered design and design thinking, emphasizing active user involvement, iterative design processes, and multidisciplinary collaboration to develop effective technological solutions through different techniques such as participatory design and rapid prototyping [38,39]. The adoption of UCD approaches for developing digital solutions in older adults has been extensively explored [40-43] and is even more crucial for individuals with cognitive decline [44-46]. Standard design approaches often fall short when developing interventions for these populations because they fail to account for specific cognitive limitations and needs, reduced technology familiarity, and unique user contexts [47-49]. UCD addresses these challenges through structured, iterative cycles that follow 4 key stages: understanding the context of use, specifying user requirements, producing design solutions, and evaluating designs against requirements. When applied to older adults, as suggested by Nimmanterdwong et al [49], this process requires recruiting older participants, identifying their specific needs, incorporating measurable goals, creating rapidly testable prototypes, and adapting usability techniques to accommodate age-related limitations. In particular, UCD becomes particularly relevant for home-based interventions, as these solutions should be integrated into users’ daily routines without direct professional support. Without careful consideration of user experience, even evidence-based interventions may fail due to incorrect use or poor adherence. For instance, Mehra et al [43] applied UCD methods to develop a tablet-based exercise app for older adults, revealing that iterative testing with end users was crucial for identifying unexpected interaction barriers and designing appropriate solutions.

### The DUAL-REHAB Training: Addressing Research Gaps Through 360° Technology

Despite promising advancements in technology-enhanced DT training [27], several significant research gaps remain unaddressed. First, it remains less investigated whether training gains can be effectively transferred to novel contexts [50], particularly in daily life settings. Most training interventions were conducted in laboratory settings or using commercially available video games, limiting the possibility of generalizing the acquired skills to real-life contexts [29,51]. Second, due to potential mobility and/or physical constraints, transportation limitations, or insufficient facilities in nearby communities, older adults still face significant challenges accessing cognitive training programs. From this perspective, further exploitation of technological innovations could solve the challenges associated with traditional face-to-face interventions. Third, it is crucial to note that controlled randomized trials for older adults with SMCs are rarely included in DT research [21], despite facing a higher risk of developing MCI or dementia compared to those without such disorders [52-54]. To address these research gaps, the DUAL-REHAB project [55] aimed to exploit the potential of 360° technology, which is increasingly being adopted in assessment and cognitive training contexts [56-61]. 360° video represents a particularly accessible technology, offering significant advantages over other VR-based approaches. Since 360° cameras can easily capture real-life environments, they provide a practical solution for generating immersive and realistic scenarios where older adults can actively participate in cognitive-motor exercises, thus providing enhanced opportunities for transferring skills to real-life contexts. Regarding accessibility challenges, 360° content can be easily integrated into high-end applications for in-hospital sessions with therapists. The same format could be delivered through mobile apps and tablets for home sessions, overcoming transportation and facility access barriers. The DUAL-REHAB project will investigate the efficacy of this novel approach with both SMC and MCI populations, specifically including the underrepresented group of older adults with SMCs. The program’s structure includes 10 days of training, each featuring approximately 10 DT exercises replicating real-life scenarios, implemented through both an immersive modality in clinical settings (using a head-mounted display and cycle ergometer) and a nonimmersive version for at-home use (via tablet app and portable cycle ergometer).

### Study Objectives

To ensure that the DUAL-REHAB training program meets the needs and expectations of end users, as explained before, a UCD approach was adopted throughout the design and development process. This study represents the initial phases of a comprehensive UCD process, and it was designed with two primary objectives: (1) to obtain a comprehensive understanding of the end users of the DUAL-REHAB project, specifically targeting older individuals with SMC and older adults with MCI. This involved exploring various aspects, such as their lifestyles, habits, perceived well-being, technology adoption, and their needs and expectations regarding cognitive training and technologies for cognitive-motor DT training; and (2) to obtain feedback on an initial functional prototype of the DUAL-REHAB mobile app, specifically evaluating its overall functionality from the perspective of the target end users.

By systematically incorporating user perspectives throughout the development process, this study aims to ensure that the resulting training not only addresses clinical objectives but also aligns with the capabilities, preferences, and contexts of its intended users, potentially enhancing engagement, usability, and clinical outcomes.

## Methods

### Overview

A 2-step procedure was implemented to achieve our objectives. Phase 1 (user requirements) investigated end users’ lifestyles, habits, well-being, technology adoption, and expectations regarding cognitive-motor DT training. Phase 2 (prototype evaluation) tested a DUAL-REHAB mobile app prototype with end users. We used semistructured interviews with open-ended and multiple-choice questions, and a think-aloud method to evaluate user-prototype interaction (Multimedia Appendix 1).

### Participants and Recruitment

Overall, 14 participants were initially recruited for the study. The sample included 7 women with a mean age of 79 (SD 3.79) years and 7 men with a mean age of 82.43 (SD 5.65) years. Participants were recruited from patients and outpatients of the Department of Medicine, Neurology and Rehabilitation, Istituto di Ricovero e Cura a Carattere Scientifico Istituto Auxologico Italiano, Milan, Italy. In accordance with UCD principles, we recruited participants who represent our intended end users. Researchers contacted potential participants who met the following inclusion criteria: (1) age ≥60 years (no maximum age limit) to target the demographic most affected by age-related cognitive changes, (2) normal or corrected-to-normal vision to ensure participants could adequately view and interact with the visual elements of the technology, and (3) self-reported or caregiver-reported cognitive concerns or memory complaints. Exclusion criteria were (1) debilitating medical, psychiatric, or neurological conditions that could affect performance; and (2) severe cognitive impairments indicated by a score <24 on the Italian version of the Mini-Mental State Examination [62,63].

To determine participants’ cognitive status and establish diagnostic categories, an expert neuropsychologist conducted a comprehensive assessment using a battery of Italian-validated tests, including:

1. The Addenbrooke’s Cognitive Examination III [64]
2. The forward version of Digit Span and Corsi Span [65]
3. The phonemic/semantic alternate fluency test [66]
4. The Short-Story Recall and Rey figure task [67]
5. The Frontal Assessment Battery [68]
6. The Trail Making Test [69]

These neuropsychological tests were used only for diagnostic purposes and were not used for analyses in any other part of the study.

MCI participants were identified based on self-reported or caregiver-reported cognitive decline, objective impairment in one or more cognitive domains, preserved functional abilities, and absence of dementia diagnosis [70,71], as documented in hospital medical records. SMC participants were identified based on self-reported memory complaints without objective cognitive decline [52], assessed through an ad hoc qualitative interview using established criteria [72].

Of the 14 initially recruited participants, 13 met the criteria for inclusion: 8 participants (3 women and 5 men) were categorized as MCI, and 5 participants (3 women and 2 men) as SMC. One female participant was excluded because she did not meet the criteria for either group.

### Materials and Procedure

#### Phase 1: User Requirements

A semistructured interview with both open-ended and multiple-choice questions was developed specifically for this study to address the research objectives of the DUAL-REHAB project. Following UCD principles [34,35], both open-ended and multiple-choice questions were developed based on literature on technology acceptance in older adults [47,48], cognitive training preferences [23-26], and UCD approaches for older adults with cognitive impairments [44-46]. The content was subsequently reviewed by the multidisciplinary research team, including neuropsychologists and UCD experts, specifically considering the unique needs of older adults with MCI and SMC [49]. The procedure consisted of a semistructured interview administered by 1 experimenter, with a second experimenter observing and taking notes, conducted in a research room at the Department of Geriatrics and Cardiovascular Medicine of IRCCS – Istituto Auxologico Italiano in Milan. The interview comprised 2 parts: open-ended questions exploring lifestyles, habits, and socio-demographic details, followed by a questionnaire with multiple-choice responses covering perceived well-being, digital habits and skills, cognitive training knowledge, and DT cognitive-motor training expectations. While designed for independent completion, the interviewer assisted when needed or verbally administered questions in cases of fatigue to optimize time and resources. All multiple-choice questions used 5-point Likert scales ranging from “not at all” to “very much,” “poor” to “excellent,” or “never” to “every day.” Each participant was welcomed inside the room with his or her companion, who was dismissed after explaining the procedure. The interview administration lasted between half an hour and 1 hour, depending on participants’ reading speed, comprehension, response time, and the extent of their conversational digressions. Experimenters remained flexible regarding digressions as these could provide valuable information and help participants feel comfortable.

#### Phase 2: Prototype Evaluation

For Phase 2, a prototype of the DUAL-REHAB mobile app designed for DT motor-cognitive training was developed. It included DT exercises planned over 10 days in the final version. The experimenters created a Microsoft PowerPoint presentation displayed on an 11.2-inch iPad (A1566) and administered it to the participants. The presentation slides simulated the user journey from the home screen to the screen displaying DT exercises. The prototype was meticulously designed to ensure an optimal user experience, considering elements such as graphics, colors, user-system dialogue, and text adherence to International Organization for Standardization 9241 guidelines (ISO [International Organization for Standardization] 9241-11:2018, 2018). While the prototype was primarily based on established usability guidelines, some initial considerations from Phase 1 (see Phase 1: user requirements) informed basic design decisions, such as interface simplification and clear visual hierarchy.

Participants were instructed to interact with the slides, following written instructions in a binder. The instructions directed individuals to simulate starting the app and completing exercises scheduled for the first and second training days. The instructions provided were as follows:

Simulation of the first day of rehabilitation

1. Open and enter the app.
2. Launch the first day of exercises.
3. Select the first exercise of the day.
4. Listen to the exercise instructions.
5. Launch the exercise.

Simulation of the second day of rehabilitation

1. Open and enter the app.
2. Launch the second day of exercises.
3. Select the first exercise of the day.
4. Listen to the exercise instructions.
5. Listen to the instructions again.
6. Launch the exercise.

Each exercise was designed with the option to relisten to the instructions if a specific button was selected. Participants were instructed to listen again to the instructions of the exercise, even if they understood them initially, to evaluate whether they could successfully interact with this feature when experiencing comprehension difficulties. According to the think-aloud method [73], participants were explicitly instructed to verbalize their thoughts and decision-making while interacting with the prototype. This cognitive verbalization technique requires participants to continuously “think aloud” by speaking everything that comes to mind during task completion, without filtering or interpreting their thoughts. This approach provides direct access to participants’ cognitive processes and immediate reactions to the interface, capturing usability issues and misunderstandings as they occur rather than relying on retrospective reports that might be affected by memory limitations or post-hoc rationalizations. During the observation, researchers documented participants’ verbalizations, hesitations, mistakes, and questions, along with their ability to complete tasks independently or with assistance. This real-time data collection method is particularly valuable for identifying specific interface elements that cause confusion or difficulty.

### Ethical Considerations

The study was approved by the Ethics Committee of IRCCS – Istituto Auxologico Italiano, with protocol number approval 2023_01_31_10. All participants provided informed written consent before enrollment in the study. To ensure privacy, all potentially identifying information was removed, and data were kept strictly confidential. Participants received no financial compensation.

## Results

### Participants Characteristics

Selected participants were the same for this study’s Phase 1 and Phase 2. The main socio-demographic information is presented in Table 1.

**Table 1:** Description of the characteristics of the older adults with mild cognitive impairment and subjective memory complaints (N=13).

Characteristics		Values						
**Cognitive conditions, n (%)**								
	MCI^a^						8 (61.54)	
	SMC^b^						5 (38.46)	
**Age groups (years), n (%)**								
	65-75							0 (0)
	75-80							7 (53.85)
	80-85							1 (7.69)
	85-90							5 (38.46)
**Sex, n (%)**								
	Female			6 (46.15)				
	Male				7 (53.85)			
**Marital status, n (%)**								
	Single			2 (15.39)				
	Married or common-law relationship			5 (38.46)				
	Divorced			0 (0)				
	Widowed			6 (46.15)				
**Education, n (%)**								
	Primary school diploma						0 (0)	
	Middle school diploma						4 (30.77)	
	Professional diploma						1 (7.69)	
	High school diploma						4 (30.77)	
	University degree or tertiary education						4 (30.77)	
**Occupational status, n (%)**								
	Employed					1 (7.69)		
	Unemployed					0 (0)		
	Retired					12 (92.31)		
**Living situation, n (%)**								
	Alone		5 (38.46)					
	With others	8 (61.54)						

^a^MCI: mild cognitive impairment.

^b^SMC: subjective memory complaints.

### Phase 1: User Requirements

#### Overview

This section presents findings from our semistructured interviews examining user characteristics, needs, and preferences. We systematically analyze different components of the interview data to understand potential users of the DUAL-REHAB training.

#### Lifestyle and Habits

In the initial interview phase, participants were presented with an open-ended question to investigate their lifestyle and habits: “Describe a typical day from waking up until going to bed, without unexpected or extraordinary events.”

A structured checklist guided the interviewer in systematically collecting information across multiple domains, such as family and social relationships, leisure activities, daily habits, nutritional patterns, sleep quality, and physical activity levels.

Participants typically reported waking up around 7 AM; primary meals (lunch and dinner) were predominantly prepared and consumed at home. Between meals, participants are commonly engaged in activities including walking, reading, television viewing, or resting periods. Household management tasks and fulfillment of daily responsibilities were consistently reported. In total, 5 participants specifically noted attending to health-related needs, either their own or their spouses’, through medication management or attendance at medical appointments for more intensive therapeutic interventions.

Overall, participants demonstrated adequate cognitive and motor autonomy levels for daily functioning, with occasional exceptions due to specific conditions such as temporary limitations following falls or chronic pain. While social relationships were universally identified as fundamental, many participants reported challenges maintaining social connections due to diminished friendship networks or geographical separation from family members with competing obligations. Nevertheless, 7 participants specifically identified interactions with children and grandchildren as significant protective factors for emotional well-being.

These insights informed the development of 2 distinct “personas” (Multimedia Appendices 2 and 3), a UCD technique that synthesizes representative user behaviors and needs [74].

#### Perceived Well-Being

Participants’ perception of well-being appeared moderate, as indicated by mean scores of 2.79 (SD 0.97) for physical health, 3.14 (SD 0.86) for psychological health, and 3.14 (SD 0.53) for cognitive health on a Likert scale ranging from 1 to 5.

#### Habits and Digital Skills

On a Likert scale ranging from 1 (“very bad”) to 5 (“excellent”), participants demonstrated a relatively poor relationship with technology, as evidenced by a mean score of 2.57 (SD 0.94). Moreover, their perception of personal digital literacy (the ability to interact with digital tools) was even lower, with a mean score of 2.21 (SD 0.89).

Tables 2-4 present the frequency distributions for technological assistance received, device use (tablets, smartphones, smart televisions, and PCs), and digital service use (health, shopping, administrative, and entertainment), respectively.

**Table 2:** Frequency of technological assistance received by participants (N=13).

Technological assistance received	Values (N=13), n (%)
Never	1 (7.69)
Rarely	2 (15.39)
From time to time	5 (38.46)
Often	5 (38.46)
Everyday	0 (0)

**Table 3:** Frequency of use of technological devices (N=13).

Device use frequency		Values (N=13), n (%)
**Tablet**		
	Never	7 (53.85)
	Rarely	1 (7.69)
	From time to time	1 (7.69)
	Often	2 (15.38)
	Everyday	2 (15.38)
**Laptop**		
	Never	5 (38.46)
	Rarely	0 (0)
	From time to time	3 (23.08)
	Often	0 (0)
	Everyday	5 (38.46)
**Smart-tv**		
	Never	9 (69.23)
	Rarely	0 (0)
	From time to time	0 (0)
	Often	1 (7.69)
	Everyday	3 (23.08)
**Smartphone**		
	Never	5 (38.46)
	Rarely	1 (7.69)
	From time to time	0 (0)
	Often	0 (0)
	Everyday	7 (53.85)

**Table 4:** Frequency of use of digital services (N=13).

Digital service use frequency		Values (N=13), n (%)
**Public services and administration**		
	Never	3 (23.08)
	Rarely	3 (23.08)
	From time to time	5 (38.46)
	Often	2 (15.38)
	Everyday	0 (0)
**Health services**		
	Never	10 (76.92)
	Rarely	1 (7.69)
	From time to time	1 (7.69)
	Often	1 (7.69)
	Everyday	0 (0)
**Shopping services**		
	Never	11 (84.62)
	Rarely	0 (0)
	From time to time	0 (0)
	Often	2 (15.38)
	Everyday	0 (0)
**Email and messages**		
	Never	3 (23.08)
	Rarely	3 (23.08)
	From time to time	1 (7.69)
	Often	3 (23.08)
	Everyday	3 (23.08)
**Social network**		
	Never	8 (61.54)
	Rarely	0 (0)
	From time to time	3 (23-08)
	Often	1 (7.69)
	Everyday	1 (7.69)
**News**		
	Never	10 (76.92)
	Rarely	0 (0)
	From time to time	0 (0)
	Often	2 (15.38)
	Everyday	1 (7.69)
**Audio or video streaming**		
	Never	7 (53.85)
	Rarely	1 (7.69)
	From time to time	2 (15.38)
	Often	2 (15.38)
	Everyday	1 (7.69)

Overall, findings suggested that smartphones dominated daily use, with 7 out of 13 participants (53.85%) using them every day. However, other devices show more sporadic patterns, with tablets and laptops being the least frequently used among our participants. On the other hand, it is interesting to note a strong heterogeneity across service categories. Health services showed the lowest penetration (10/13, 76.92% reported never using them), while email services, audio or video streaming, and social networks demonstrate more distributed use.

Two general evaluation parameters were considered in assessing technology: curiosity and use. While participants did not perceive themselves as particularly curious about technology (mean 2.71, SD 1.54, with 3 indicating “neutral”), they did recognize its usefulness in daily life (mean 3.57, SD 1.28).

#### Cognitive Training

Regarding cognitive training, we aimed to explore the end users’ prior knowledge of the feasibility and methodologies involved in training cognitive functions and their level of interest in this area. Twelve participants demonstrated awareness of the potential for cognitive training to improve mental health, encompassing memory, attention, and perception. Eleven individuals also acknowledged the feasibility of incorporating cognitive and motor exercises. At the same time, 12 recognized that the objective of such training could be to enhance cognitive functions or impede their decline.

Interest in improving cognitive abilities was substantial, with a mean rating of 4.15 (SD 1.34), on a Likert scale from 1 to 5. Similarly, participants perceived the personal use of cognitive training, with a mean rating of 4.15 (SD 1.28). Only 1 participant expressed no interest in cognitive training. Furthermore, an interesting finding concerned participants’ willingness to recommend cognitive training to others, with 11 individuals expressing readiness to endorse such training. Qualitative analysis of these responses revealed diverse recommendations for peers and younger generations, including children and grandchildren, underscoring the perceived value and broad applicability of cognitive training across age groups.

#### Cognitive-Motor DT Training With Technology

In implementing integrated DT cognitive-motor training programs, this section explored participants’ knowledge and expectations regarding using technology, including tablets, smartphones, and laptops.

Among the participants, 6 individuals (approximately half of the sample) were aware of the possibility of performing cognitive-motor training with the support of technological tools. Despite this limited awareness, 12 participants expressed their willingness to participate in this type of training. However, when asked if they would also perform the training independently at home, the number of willing participants decreased slightly to 11.

Those willing to engage in home-based training were then asked to envision how much time they would dedicate to such a protocol, including weekly frequency, session duration, and overall training period. The results of these 3 questions are reported in Table 5.

**Table 5:** Time willing to dedicate to cognitive-motor dual task training at home with technology (N=11).

Home training time				Values (N=11), n (%)
**Days per week**				
	1 day	1 (9.09)		
	2-3 days	7 (63.64)		
	3-4 days	3 (27.27)		
	5-6 days	0 (0)		
	7 days	0 (0)		
**Time per day**				
	10-15 minutes	4 (36.36)		
	15-20 minutes	4 (36.36)		
	20-30 minutes	1 (9.10)		
	30-45 minutes	0 (0)		
	45-60 minutes	2 (18.18)		
**Overall duration**				
	1 week		0 (0)	
	2 weeks		4 (36.36)	
	3-4 weeks		3 (27.28)	
	5 weeks		0 (0)	
	6 weeks		4 (36.36)	

Overall, participants showed moderate willingness to dedicate time to home-based DT training. While daily and weekly commitment tended to range from moderate to low, the total duration indicated considerable availability, ranging from medium to high. Further analysis revealed that all 11 older adults would engage in the training for a maximum of 4 days per week. A total of 9 individuals would participate for 30 minutes daily, with the other 2 willing to extend it to 60 minutes. Additionally, 7 participants would undergo training for 2 to 4 weeks, with 4 participants willing to extend it to 6 weeks.

Overall, participants perceived using technology or performing cognitive-motor training with DT exercises as very useful (mean 4.31, SD 0.85) and moderately simple (mean 3.31, SD 1.38). We explored the participants’ positive expectations and primary concerns regarding a technology-supported cognitive-motor training protocol. The summarized results are presented in Tables 6 and 7. As participants could provide multiple responses, the total frequencies may exceed the number of interviewees.

**Table 6:** The most common positive expectations regarding a cognitive-motor training protocol using technological tools at home (N=13).

Positive expectations	Values (N=13), n (%)
Enhanced autonomy	8 (61.54)
Improved self-esteem and self-efficacy	6 (46.15)
Cognitive function enhancement	11 (84.61)
Improved physical health status	3 (23.08)
Enhanced self-care capabilities and awareness	6 (46.15)
Reduced frequency of medical consultations	0 (0)
Improved social interaction	1 (7.69)
Time efficiency benefits	3 (23.08)
Financial benefits	2 (15.39)
Improved daily routines and lifestyle	2 (15.39)
None of the previous answers	0 (0)
Other	0 (0)

**Table 7:** The most common concerns regarding a cognitive-motor training protocol using technological tools at home (N=13).

Concerns	Values (N=13), n (%)
Privacy concerns	5 (38.46)
Adaptation to new routines	2 (15.39)
User errors in technology operation	8 (61.54)
Technical difficulties with devices	7 (53.85)
Exercise adherence challenges	4 (30.77)
Increased stress levels	3 (23.08)
Training requirements	4 (30.77)
Need for technical assistance	5 (38.46)
None of the previous answers	1 (7.69)
Other	0 (0)

### Phase 2: Prototype Evaluation

Using an observation grid during prototype evaluation, all verbalizations were documented, including (1) comments, (2) complaints, or (3) direct requests for help, as well as observable behaviors such as (1) hesitations or (2) errors that indicated the participant’s inability to complete the tasks independently. During the data collection, only inputs regarding the system characteristics were documented.

Data collected for each participant were compared to identify tasks causing the most significant difficulties. For each critical task, we determined whether others shared similar challenges. Identifying the most problematic tasks and system characteristics would provide valuable guidance for modifications in the final app version. This comparative analysis is represented in Table 8, which specifies the most critical tasks (and their instructions), synthesizes issues preventing task completion, indicates the number of participants encountering each difficulty, and presents potential solutions.

**Table 8:** Most critical tasks and respective instructions, problems, and solutions for the think-aloud protocol of the DUAL-REHAB prototype in mild cognitive impairment and subjective memory complaints participants.

Instructions, critical tasks, and problems				Solutions		Participants, n
**Open the app**						
	**Device interaction**					
		Excessive pressure during screen interaction	Implementation of device familiarization sessions for participants without prior touchscreen experience		1	
		Difficulty identifying the app icon	Elimination of extraneous visual elements from the home screen interface		3	
	**Execution of instructions**					
		Protocol execution only following researcher’s prompting	Development of an interactive tutorial for home screen navigation		1	
**Launch day 1**						
	**Execution**					
		Day selection errors due to participants’ tendency to search for calendar dates rather than ordinal day numbers in the training sequence	Consideration of replacing numerical day identifiers with calendar dates to align with participants’ expectations		1	
	**Proper interaction with the device**					
		Attentional dispersion due to interface complexity (eg, several buttons on the screen layout)	Reduction of interface elements to minimize cognitive load		1	
**Launch day 2**						
	**Comprehension of the instructions**					
		Erroneous day selection	Provision of a tutorial or additional information on the appropriate protocol sequence navigation across different training days		1	

## Discussion

### Principal Findings

The DUAL-REHAB project represents an innovative approach to cognitive-motor rehabilitation that exploits the potential of 360° technology to develop a novel DT training program specifically designed for older adults with SMC and MCI. Following UCD principles, this study revealed important insights about our target users and prototype functionality. Participants demonstrated high autonomy in daily activities and strong motivation to maintain independence despite reduced social interactions. They reported moderate satisfaction with their well-being across physical, psychological, and cognitive domains, with physical health rated lower than cognitive health. Participants expressed strong belief in cognitive training’s potential and high interest in enhancing cognitive functions, with expected outcomes primarily focused on autonomy and self-care improvements.

Despite low self-reported technology proficiency, they showed a high willingness to participate in technology-based interventions and perceived such tools as valuable for cognitive-motor training. Participants preferred moderate training frequency (2-3 days/week) and shorter sessions (10-20 minutes). The prototype evaluation revealed some usability issues, with challenges primarily related to basic tablet interaction and navigation understanding. Key design improvements needed include simplifying the interface, redesigning icons, and implementing familiar temporal references for training day selection.

One of the main risks associated with an aging population and increased life expectancy is that older people may lose their autonomy and independence [75], requiring more assistance for longer periods. However, the target population we interviewed demonstrated a high degree of autonomy in daily activities and a strong desire to maintain independence, care for themselves, and improve their well-being. Participants maintained structured daily routines, primarily self-managed their meals, and engaged in various daily activities. This high level of autonomy could benefit the DUAL-REHAB training program, potentially facilitating the implementation of at-home training sessions where participants will access a nonimmersive version of the DT exercises via a tablet app combined with a portable cycle ergometer.

Despite this autonomy, a deeper look into participants’ lifestyles showed fewer social interactions than expected, with boredom and loneliness frequently mentioned as negative aspects of their lives. This aligns with previous research identifying social isolation as a significant risk factor for cognitive decline [76]. Interestingly, health maintenance emerged as a meaningful activity for many participants, often becoming time-consuming but purposeful. This finding suggests that framing the DUAL-REHAB program as a health-promoting activity could leverage existing motivational patterns to enhance compliance. Given that 7 participants specifically identified interactions with children and grandchildren as protective factors for emotional well-being, and 5 participants were already managing health-related needs within their family context, the training program could capitalize on these existing dynamics by incorporating caregivers as “active supporters.” This approach would potentially address both training goals and social connection needs simultaneously. For our target populations specifically, family members should serve not merely as technical support but as cognitive scaffolding, a design consideration that might extend beyond the general principle for designing technologies for older adults.

Participants’ self-perceptions of well-being deserve careful attention. Despite meeting clinical criteria for MCI or SMC, they reported moderate satisfaction with their cognitive health. Participants reported moderate levels across physical, psychological, and cognitive health domains when assessing their perceived well-being. Even if everyone included in the study is categorized according to MCI or SMC criteria, they feel quite satisfied with their cognitive well-being. It can be interpreted in 2 ways: a lack of self-awareness [77] or psychological acceptance that cognitive decline is inevitable. Notably, participants rated their physical well-being lower than their cognitive health, suggesting potential opportunities for interventions addressing both domains simultaneously, as the DUAL-REHAB approach aimed. Another aspect worth mentioning is their strong belief in the potential of cognitive training. Additionally, participants acknowledged the existence and the potential of motor-cognitive training. Consistently, they also expressed great interest in enhancing their cognitive functions. When discussing expected positive outcomes, our sample most frequently mentioned self-care-related aspects: autonomy, improvement in self-efficacy, cognitive improvements, self-awareness, and self-care.

Technology is the most delicate aspect investigated in this study. While participants reported low technology proficiency, digital literacy, and limited awareness of technology-based cognitive training possibilities, they simultaneously demonstrated high willingness to participate in such interventions (12 out of 13 participants). This apparent contradiction is coupled with their high perceived usefulness of technology for cognitive-motor training, suggesting that older adults’ limited technology use likely might derive from motivational barriers rather than a lack of interest, in line with Lee and Coughlin [47], who established perceived value as a key factor in technology adoption. These findings suggest the opportunity to frame technology as a mean to maintain cognitive health could provide sufficient motivation to overcome initial resistance, a strategy supported by Wildenbos et al [48], who found that health-related technologies achieve higher adoption rates when they address specific health concerns valued by older adults.

Finally, it is interesting to highlight that the specific training preferences expressed by participants, favoring moderate frequency (2-3 days/week) and shorter sessions (10-20 minutes), align with typical training guidelines for older adults. These parameters should inform the DUAL-REHAB implementation strategy, as they represent a self-identified sustainable level of engagement that could optimize adherence while minimizing the burden on participants.

Regarding mobile app prototype and tablet use, testing revealed fewer problems than anticipated despite the participants’ limited technological skills. Most issues were related to interaction with the tablet touchscreen (eg, pressure calibration), suggesting that the prototype design achieved appropriate simplicity for the target population. A brief training session on tablet interaction fundamentals could effectively address these basic usability barriers.

The 2 key navigation challenges emerged that require design modification. First, participants struggled to recognize the app icon, a finding consistent with research showing that older adults often have difficulty with abstract icons [78]. This necessitates redesigning icons and reducing competing visual elements on the home screen. Second, and more notably, participants had trouble selecting the correct training day, frequently defaulting to “day 1” when attempting subsequent sessions. This conceptual navigation barrier suggests that sequential numbering systems may be cognitively demanding for this population, particularly for individuals with MCI and SMC who may experience episodic memory difficulties and challenges with abstract sequencing. This supports the implementation of familiar temporal references (calendar dates) rather than abstract ordinal numbering. These findings inform some critical design decisions for the DUAL-REHAB: (1) implementing intuitive navigation with familiar conceptual models that accommodate cognitive limitations, and (2) developing a comprehensive initial training phase focusing on basic tablet interaction skills tailored to the cognitive profile of end users.

Finally, the personas developed from our interview data (Multimedia Appendices 2 and 3) will guide future design decisions for the DUAL-REHAB app. These personas reflect key patterns from our study: participants showed technological curiosity alongside dependency concerns, strong motivation for health maintenance, and clear preferences for structured training approaches. The personas will inform design decisions, such as balancing user independence with appropriate support features and involving family members as cognitive support rather than just technical help.

### Conclusions

In conclusion, this study successfully addressed its primary objectives by adopting a UCD approach for the development of DUAL-REHAB training. We used 2 methods (a semistructured interview and the think-aloud method) to obtain valuable insights into the lifestyles, habits, and technological needs of older adults with MCI and SMC, as well as crucial feedback on the initial prototype of the DUAL-REHAB mobile app. Our findings will inform the further design and development of the training program, ensuring alignment with user expectations and technological capabilities. A critical need emerged for simplifying technological processes with intuitive and user-friendly interfaces. The importance of a comprehensive pretraining or familiarization phase to prepare participants for using the app was also highlighted. Furthermore, the necessity to consider and monitor participants’ engagement and motivation levels throughout the training became evident. Sociality, well-being, and self-care were key areas for implementing and framing the training. Establishing opportunities for interaction (in particular, with caregivers and family members) during clinical sessions and at-home training could help maintain motivation, promptly solve potential problems, and satisfy participants’ need for social engagement. This approach could effectively address the participants’ desire for social interactions and feeling supported in managing their well-being.

However, this study has several limitations. First, we did not collect data about the app from experts or caregivers [79]. Future studies should incorporate such feedback to comprehensively evaluate the DUAL-REHAB app’s functionality and end users’ expectations and lifestyles. This is particularly important considering the social needs that emerged from our findings. Moreover, our sample size represents a methodological limitation. While we followed established usability testing guidelines suggesting that 4-5 participants can identify most major usability issues [80,81], the specific cognitive characteristics of our population warrant consideration. Participants with MCI and SMC may experience varying cognitive difficulties that affect their technology interaction patterns, potentially requiring larger samples to capture the full spectrum of user experiences. Individual usability issues were often identified by small numbers of participants (n=1-3), but these findings align with established usability principles where even single-user observations can reveal critical design flaws. However, we recognize that the cognitive heterogeneity within our target populations limits the generalizability of these specific findings, and larger-scale usability testing would be essential to establish the prevalence and priority of identified issues before final implementation. In addition, it is crucial to note that although the semistructured interview protocol was developed based on existing literature and expert review, the absence of pilot testing represents another limitation. To address potential comprehension issues, interviewers assisted when participants encountered difficulties or fatigue, for example, by providing clarification or reading the multiple-choice questions aloud. Moreover, they allowed for spontaneous digressions that could yield valuable insights. Finally, another limitation of our usability testing regards the use of an initial prototype created with Microsoft PowerPoint rather than a fully functional application. This solution might have affected the ecological validity of our findings, and future testing of DUAL-REHAB solutions should involve more advanced technological prototypes, including, for example, interactive elements, to fully capture potential difficulties in interacting with the training application.

By addressing these limitations and building on the insights gained, future iterations of the DUAL-REHAB project can better address the needs of older adults with MCI and SMC, potentially improving their cognitive function and overall quality of life. Integrating UCD principles throughout development will allow a more effective and engaging cognitive training program tailored to its target users’ unique needs and capabilities.

## Appendix

Multimedia Appendix 1 Microsoft Power Point presentation of the paper prototype of the DUAL-REHAB application, administered to potential end users.

Multimedia Appendix 2 Personas that summarizes male users' prototypical behaviors and needs.

Multimedia Appendix 3 Personas that summarizes female users' prototypical behaviors and needs.

## Abbreviations

DT: dual-task
ISO: International Organization for Standardization
MCI: mild cognitive impairment
SMC: subjective memory complaint
UCD: user-centered design
VR: virtual reality
