# Supplementary material for: 360°-Based Cognitive-Motor Training System for Older Adults With Cognitive Impairment: User-Centered Design and Evaluation Study
Source: JMIR Aging. 2026 Mar 13;9:e68032. doi: 10.2196/68032 (PMC13032090; doi:10.2196/68032)
Supplement: Multimedia Appendix 1 [file aging_v9i1e68032_app1.pptx]

## Slide 1
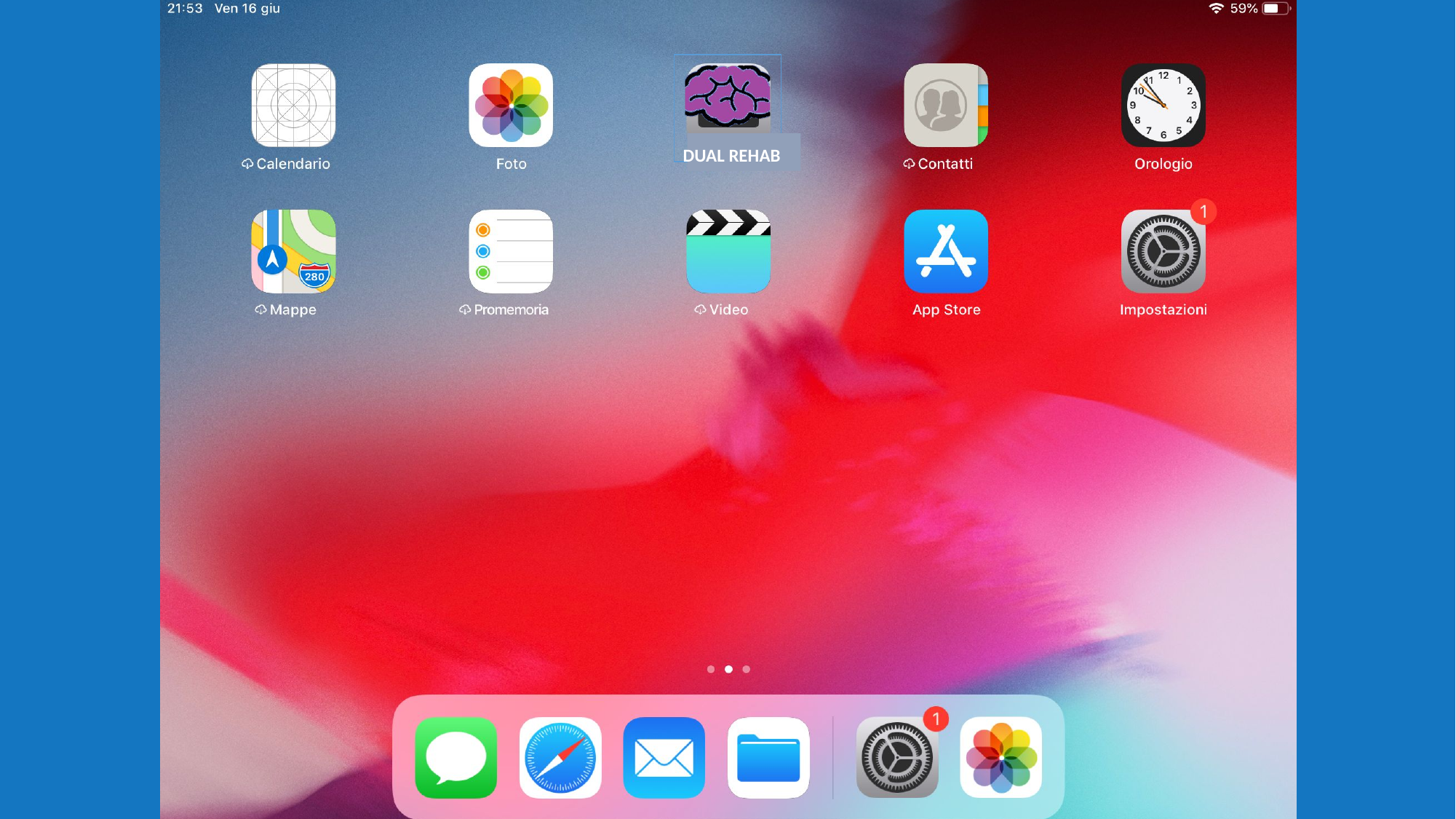

# DUAL REHAB

## Slide 2
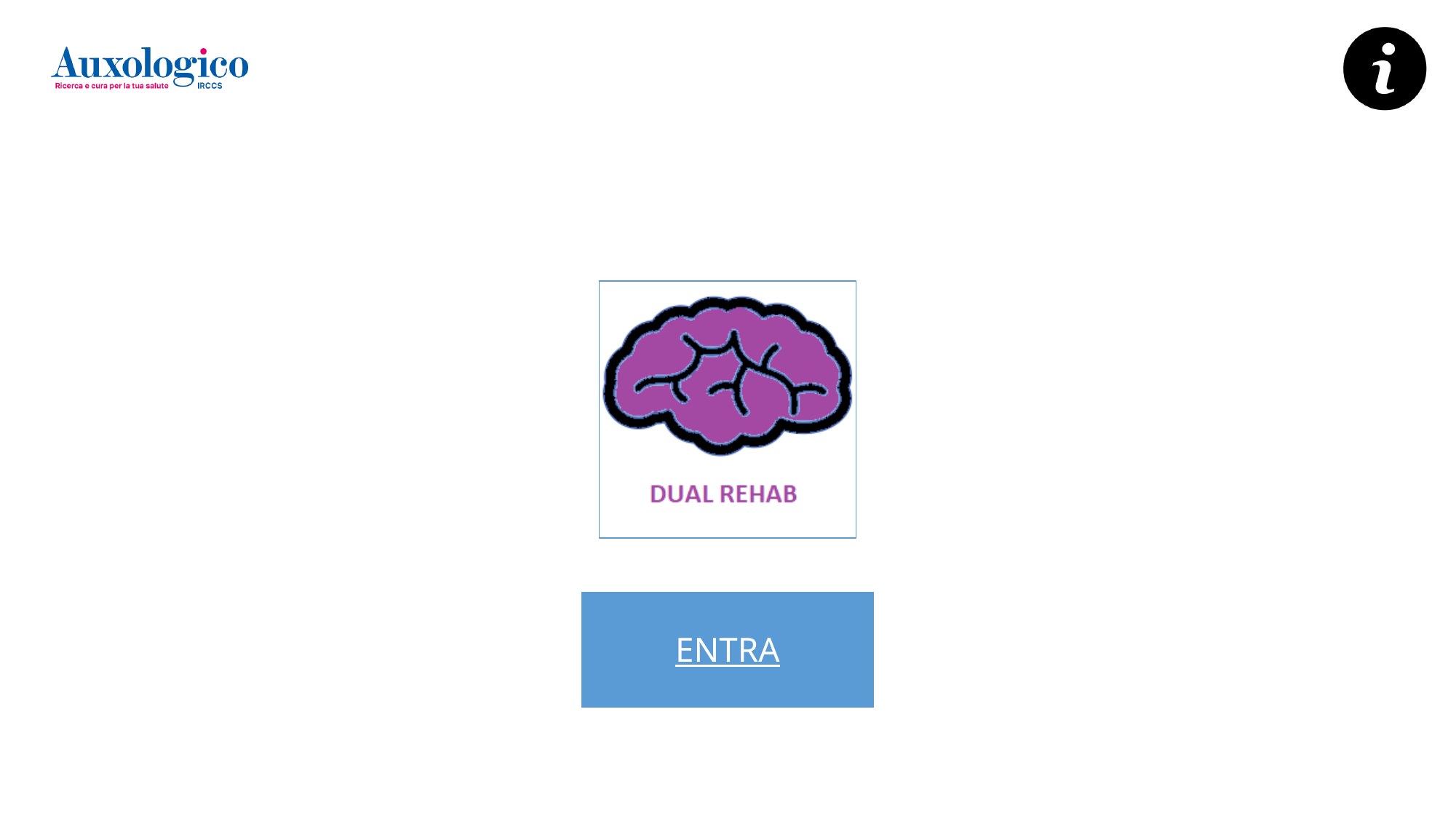

ENTRA

## Slide 3
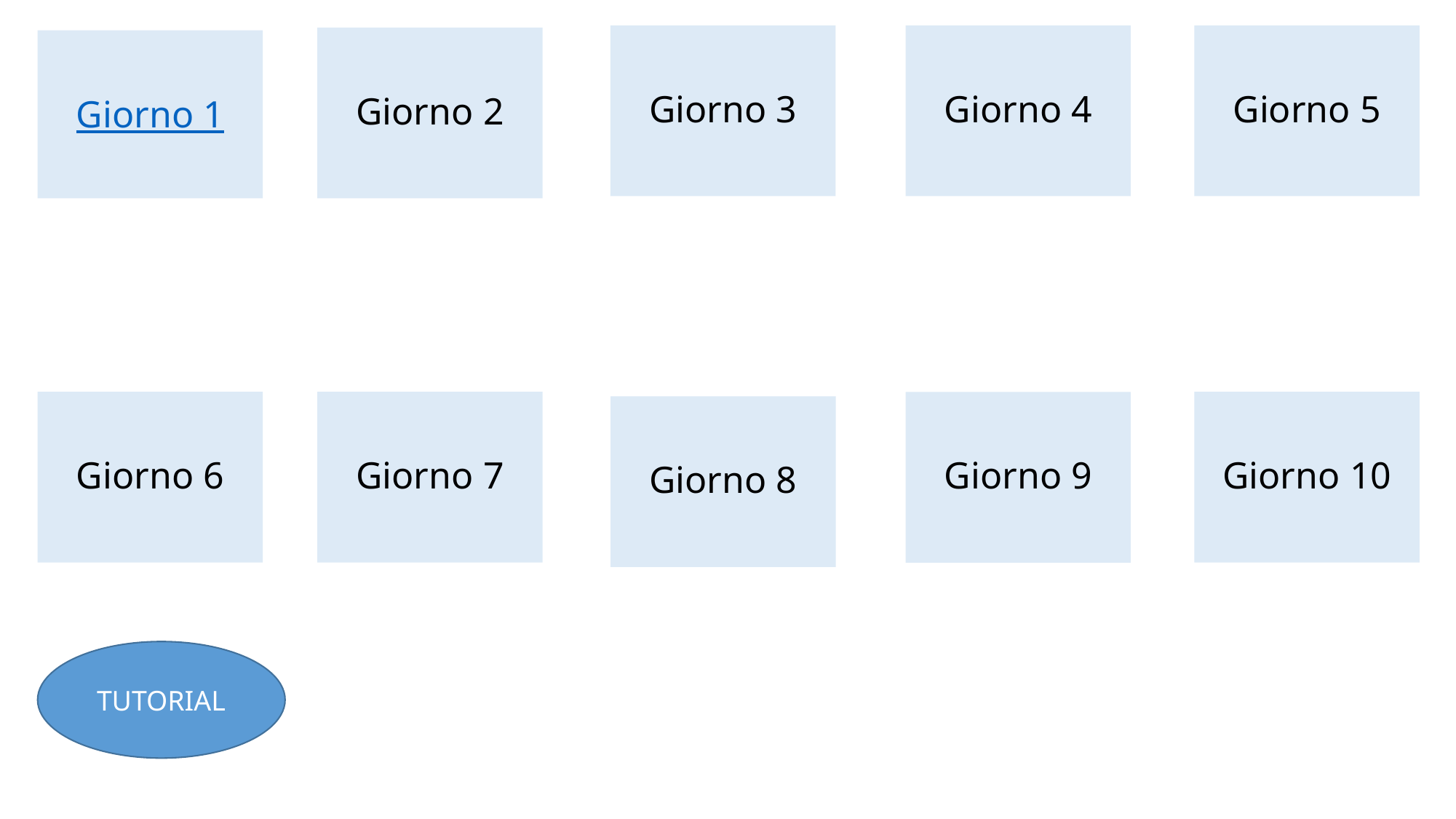

Giorno 3
Giorno 4
Giorno 5
Giorno 2
Giorno 1
Giorno 6
Giorno 7
Giorno 10
Giorno 9
Giorno 8
TUTORIAL

## Slide 4
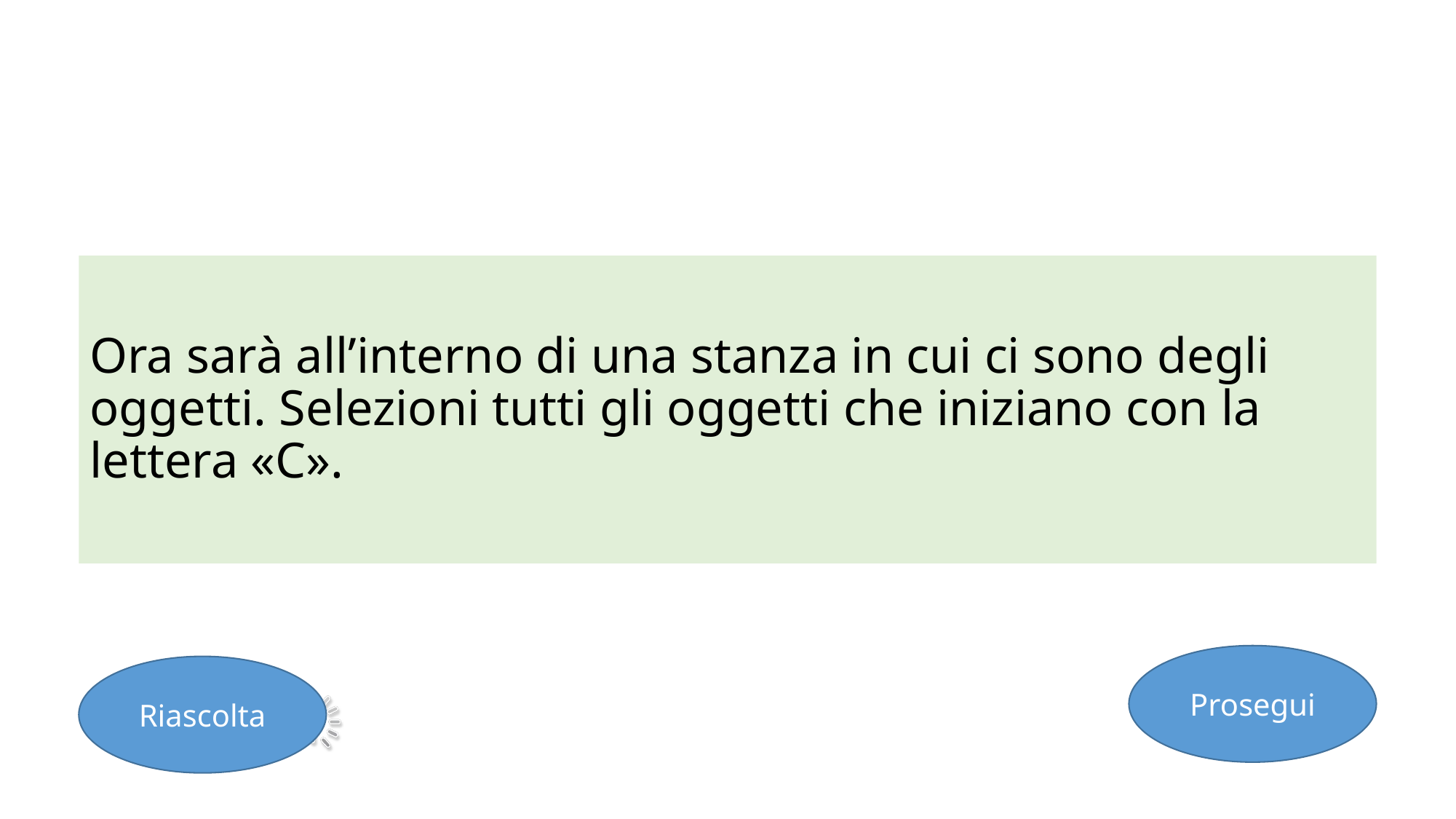

# Ora sarà all’interno di una stanza in cui ci sono degli oggetti. Selezioni tutti gli oggetti che iniziano con la lettera «C».
Prosegui
Riascolta

## Slide 5
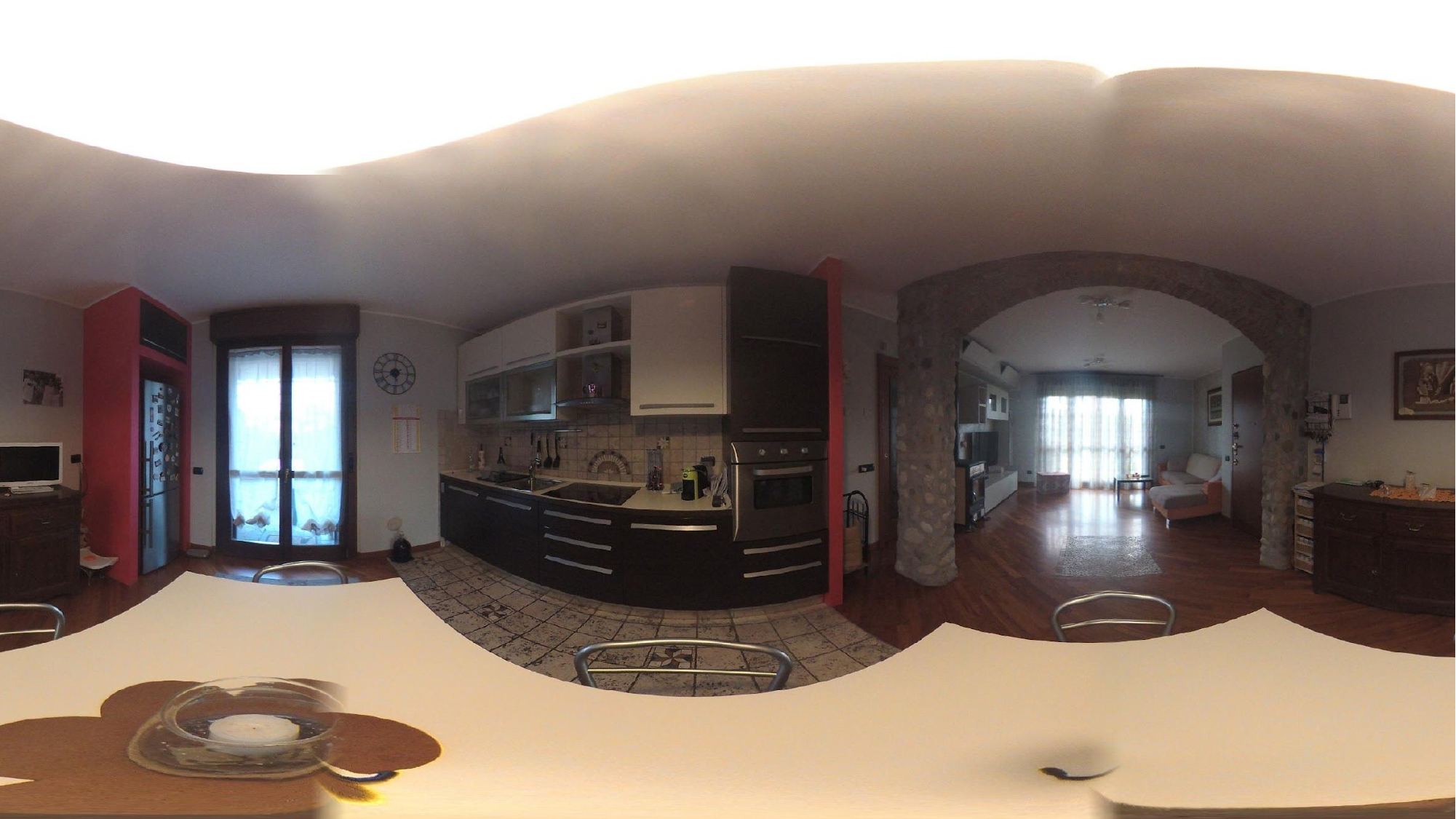

#

## Slide 6
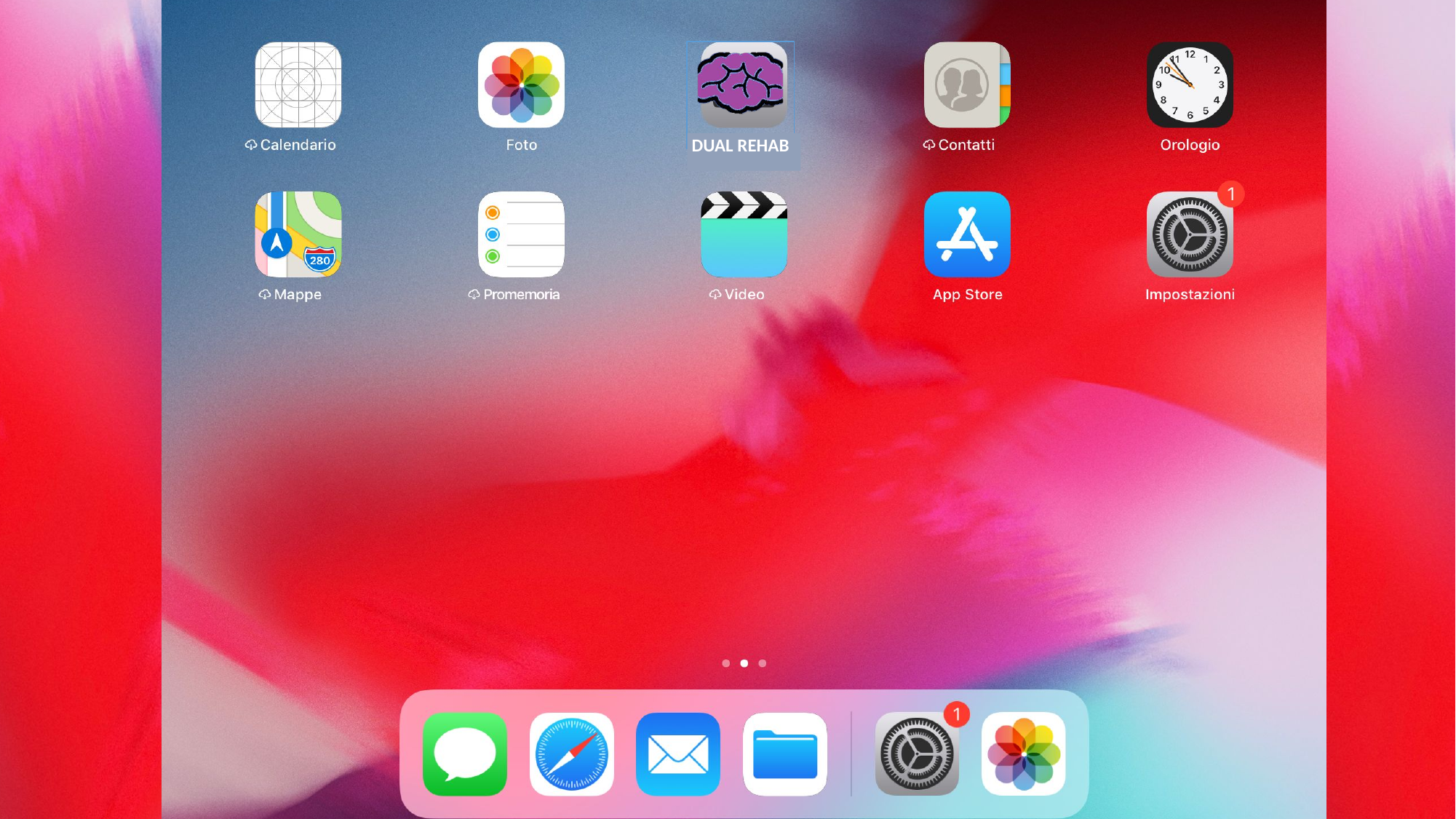

# DUAL REHAB

## Slide 7
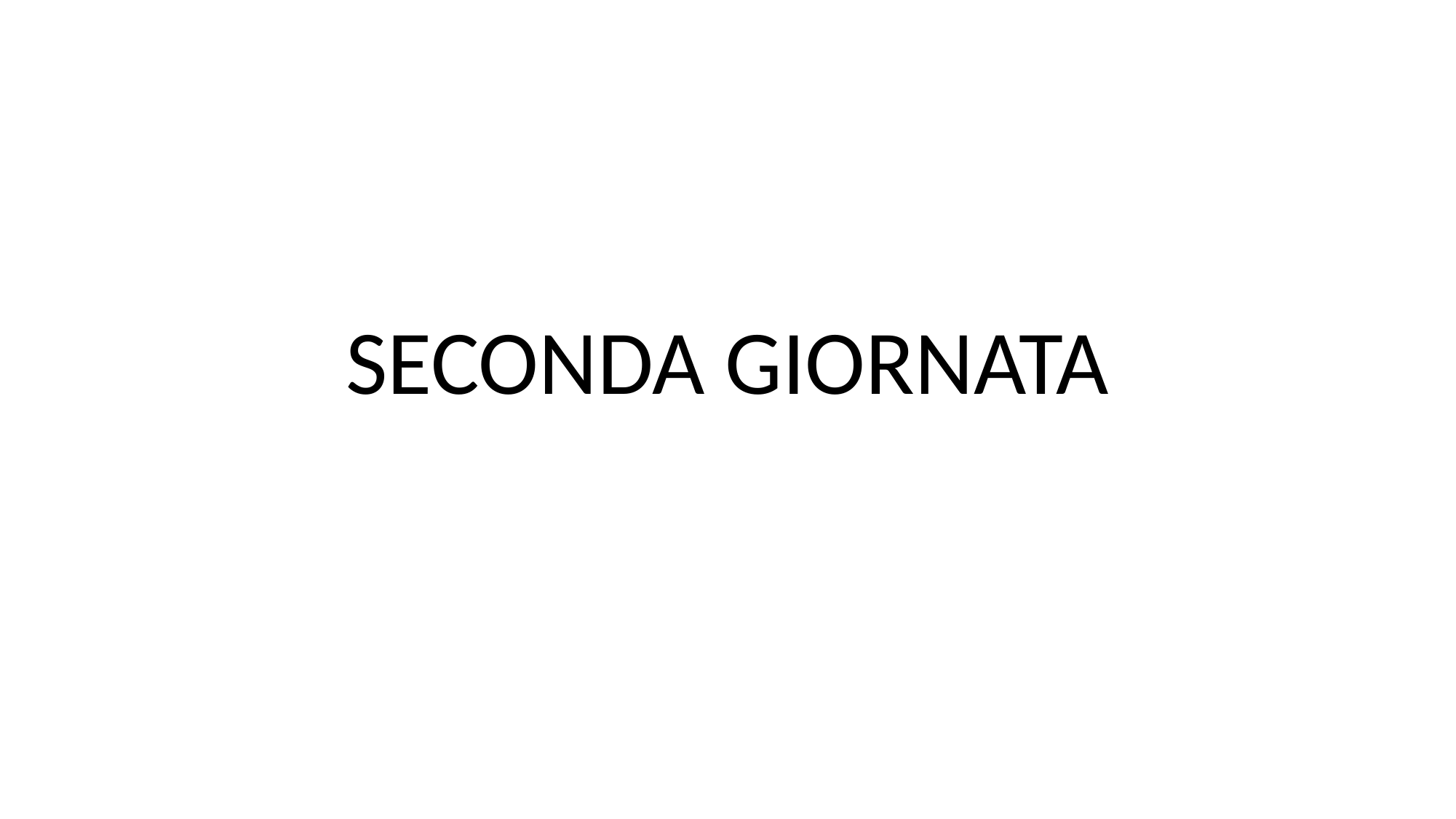

# SECONDA GIORNATA

## Slide 8
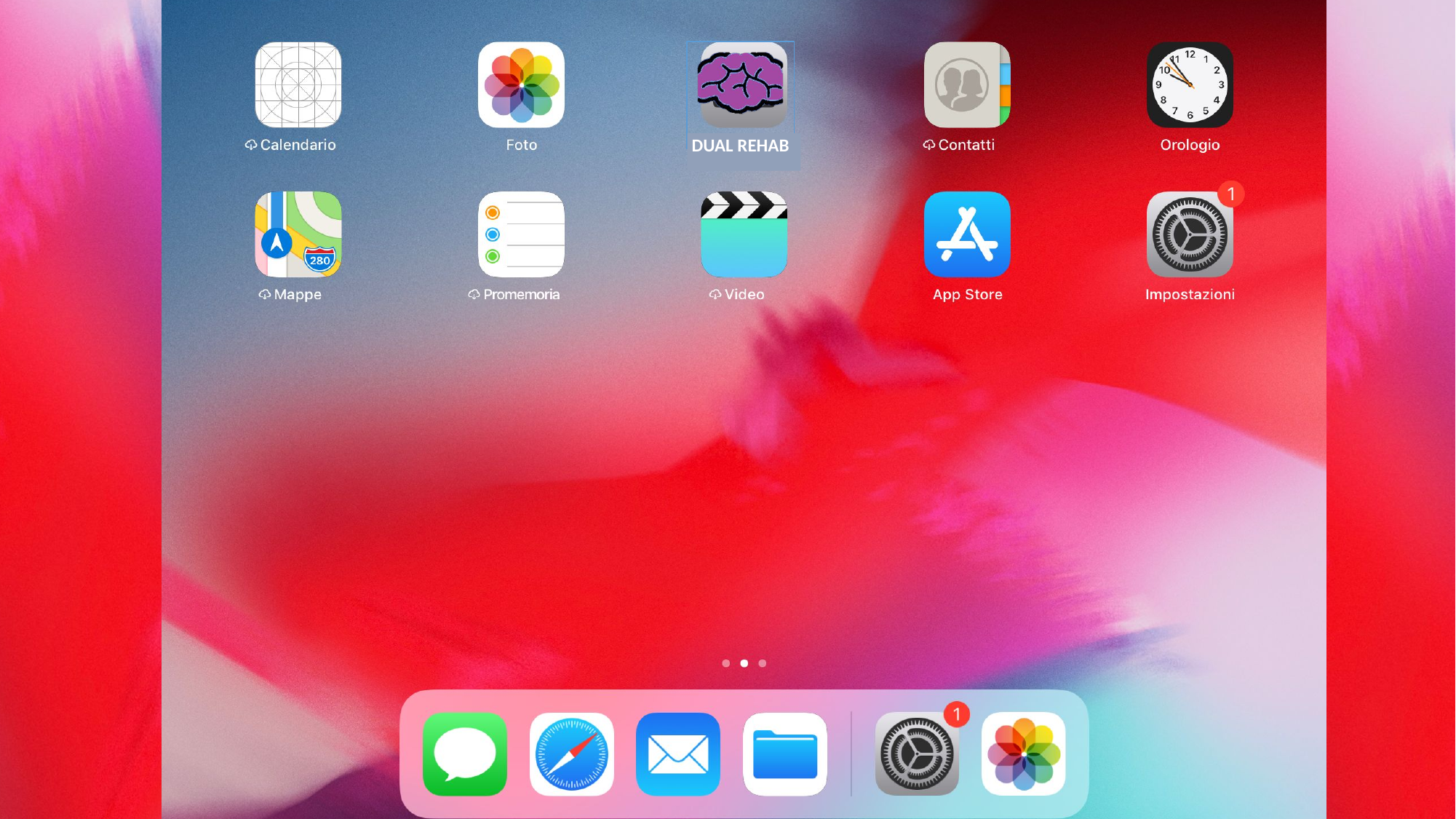

# DUAL REHAB

## Slide 9
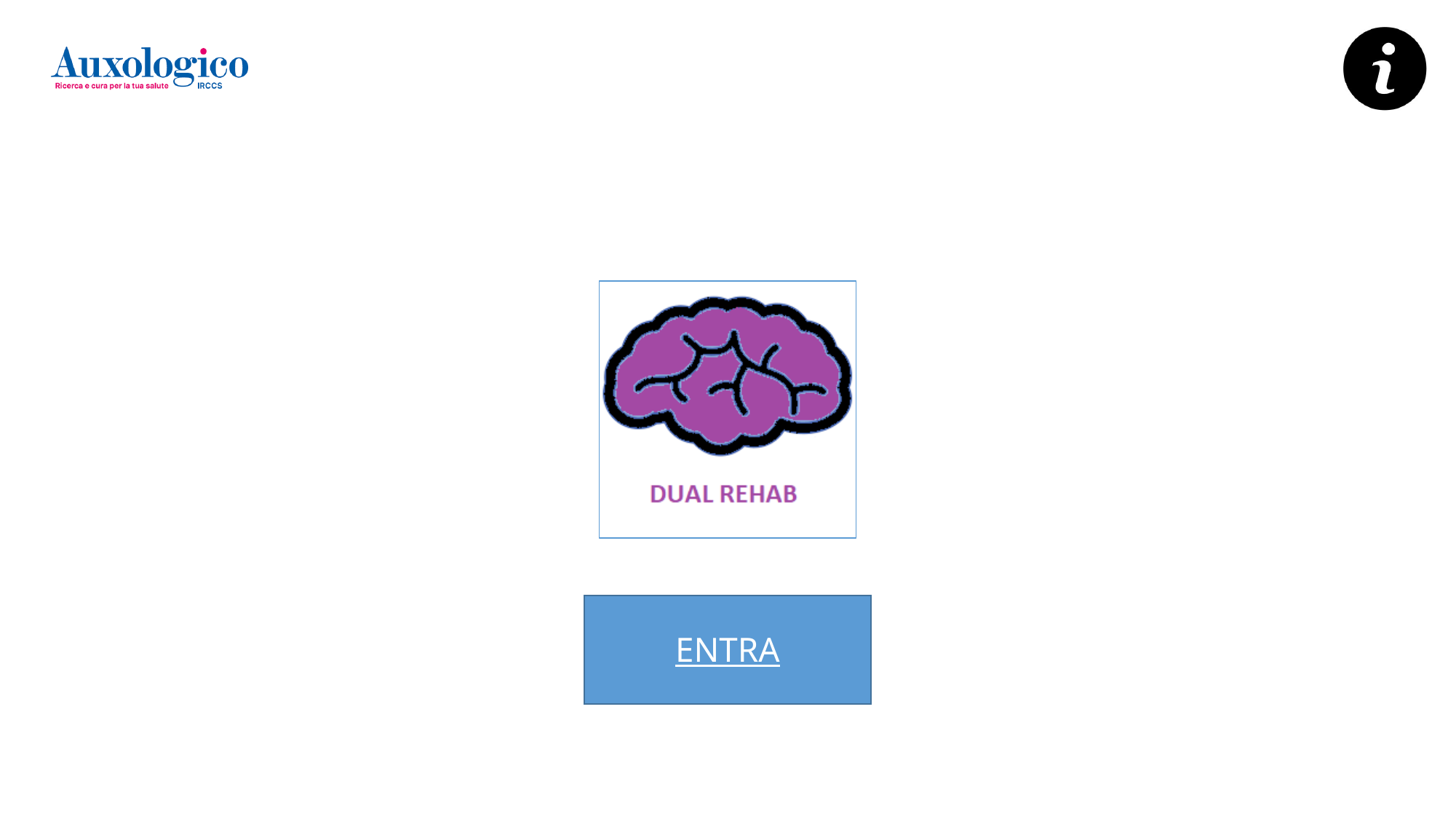

ENTRA

## Slide 10
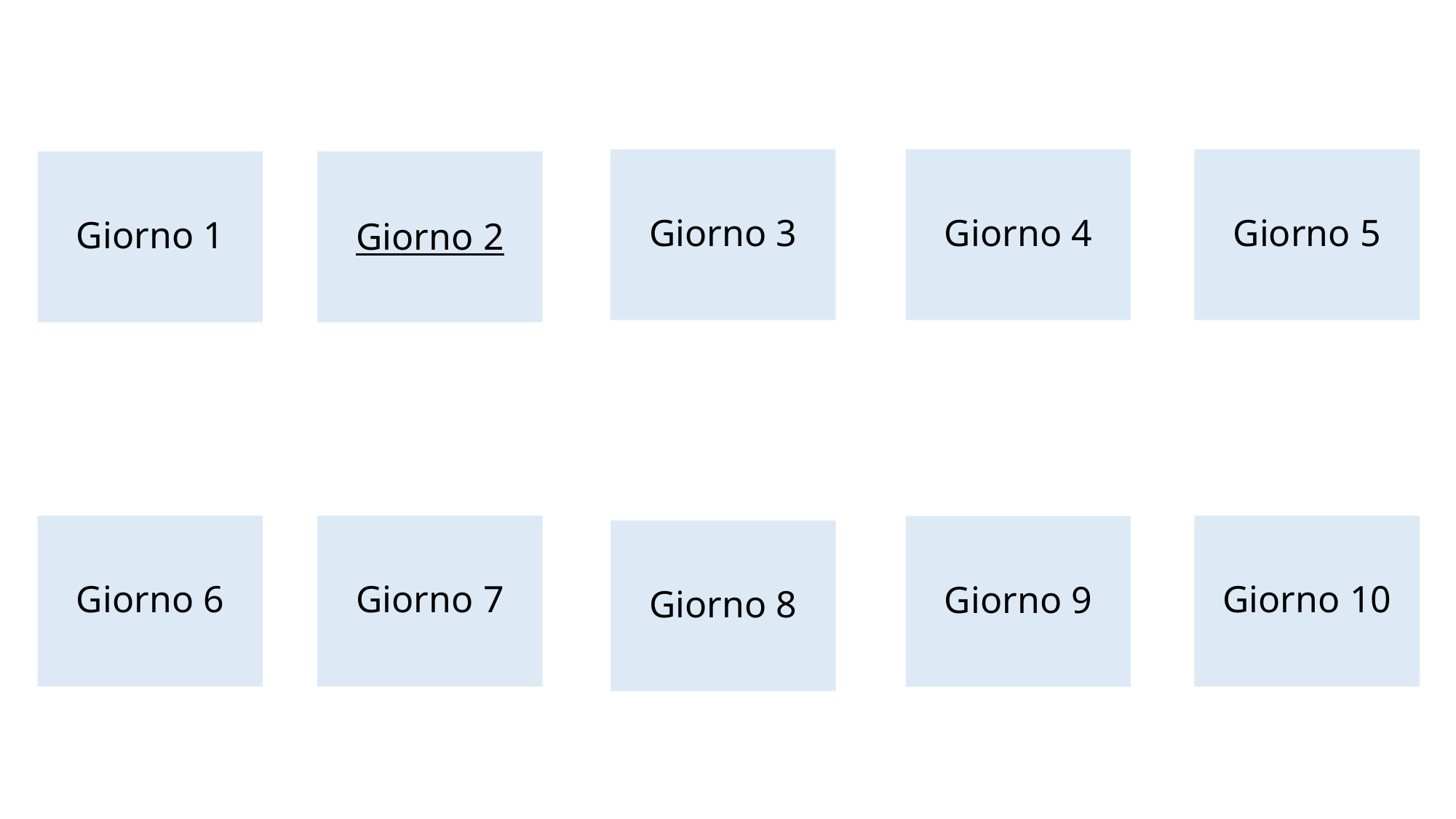

Giorno 3
Giorno 4
Giorno 5
Giorno 1
Giorno 2
Giorno 6
Giorno 7
Giorno 10
Giorno 9
Giorno 8

## Slide 11
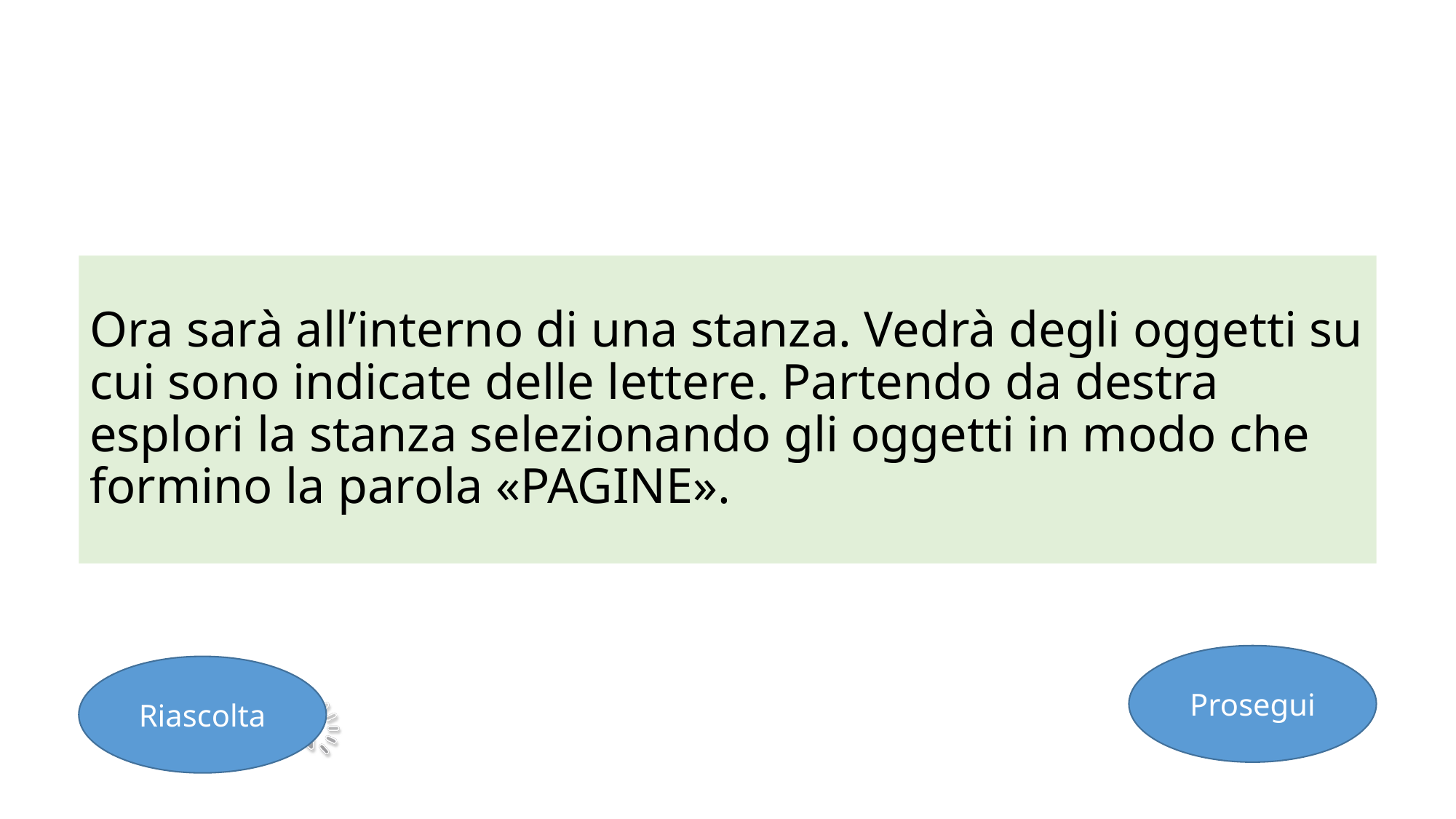

# Ora sarà all’interno di una stanza. Vedrà degli oggetti su cui sono indicate delle lettere. Partendo da destra esplori la stanza selezionando gli oggetti in modo che formino la parola «PAGINE».
Prosegui
Riascolta

## Slide 12
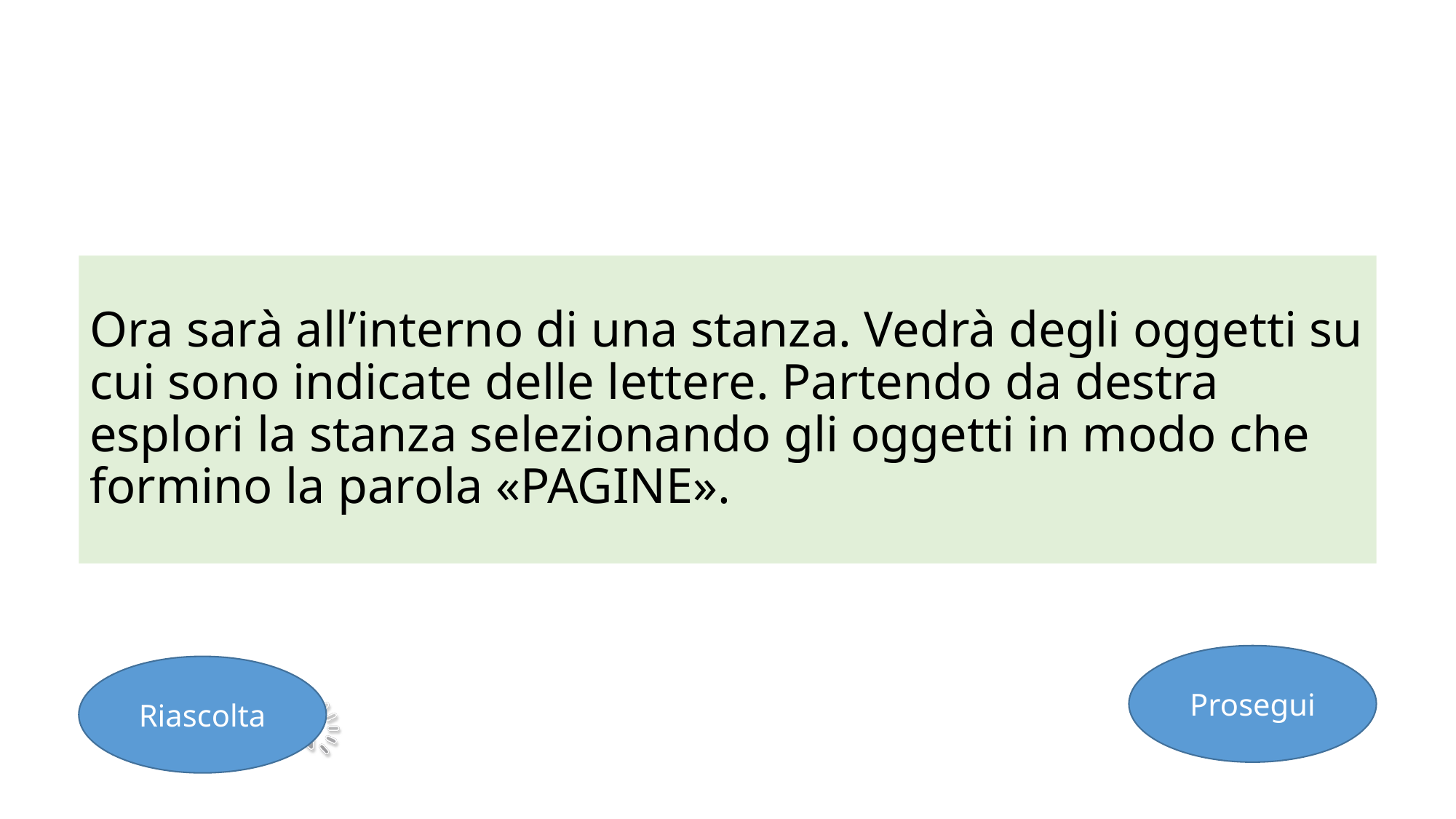

# Ora sarà all’interno di una stanza. Vedrà degli oggetti su cui sono indicate delle lettere. Partendo da destra esplori la stanza selezionando gli oggetti in modo che formino la parola «PAGINE».
Prosegui
Riascolta

## Slide 13
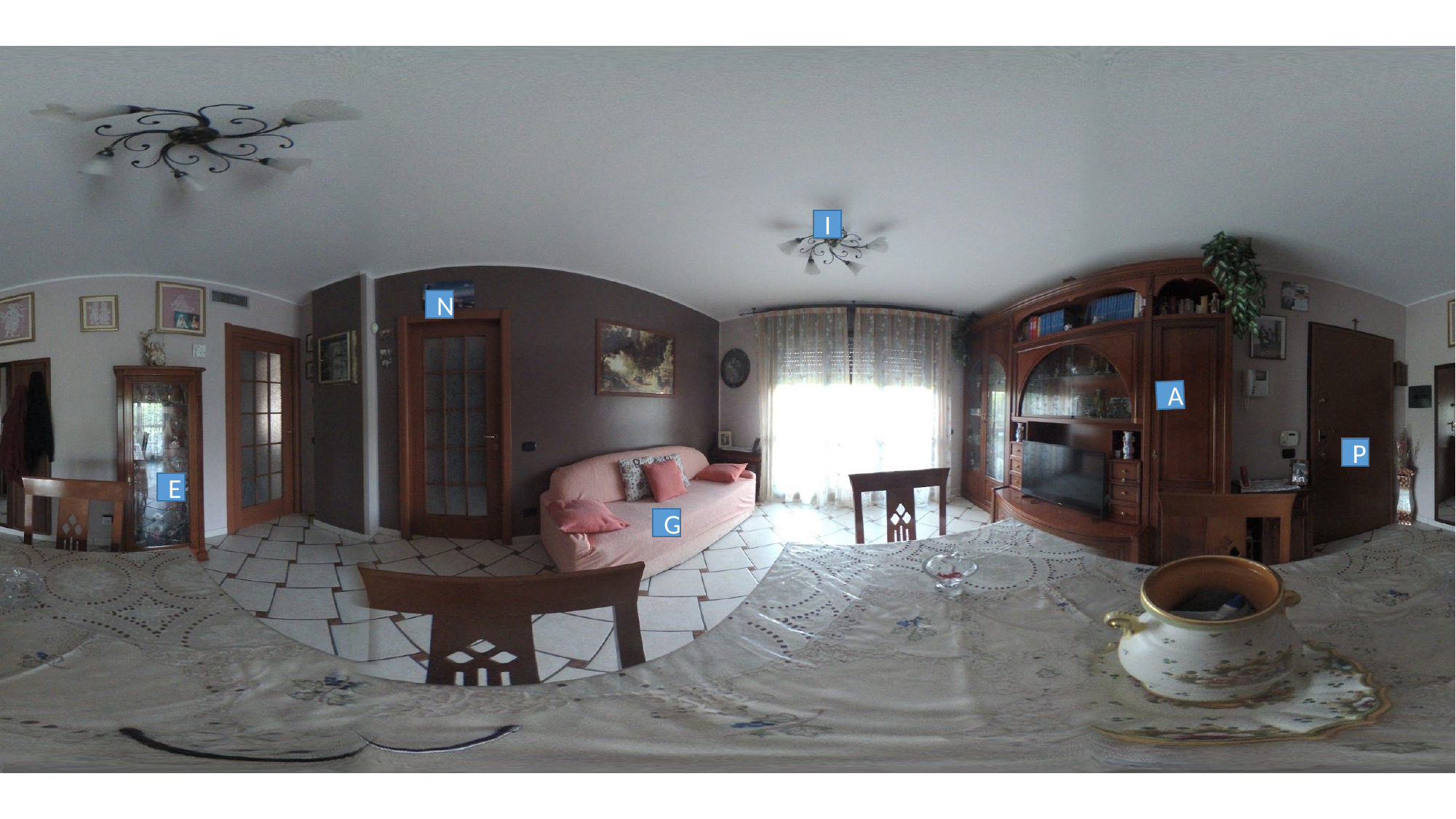

#
I
N
A
P
E
G

## Slide 14
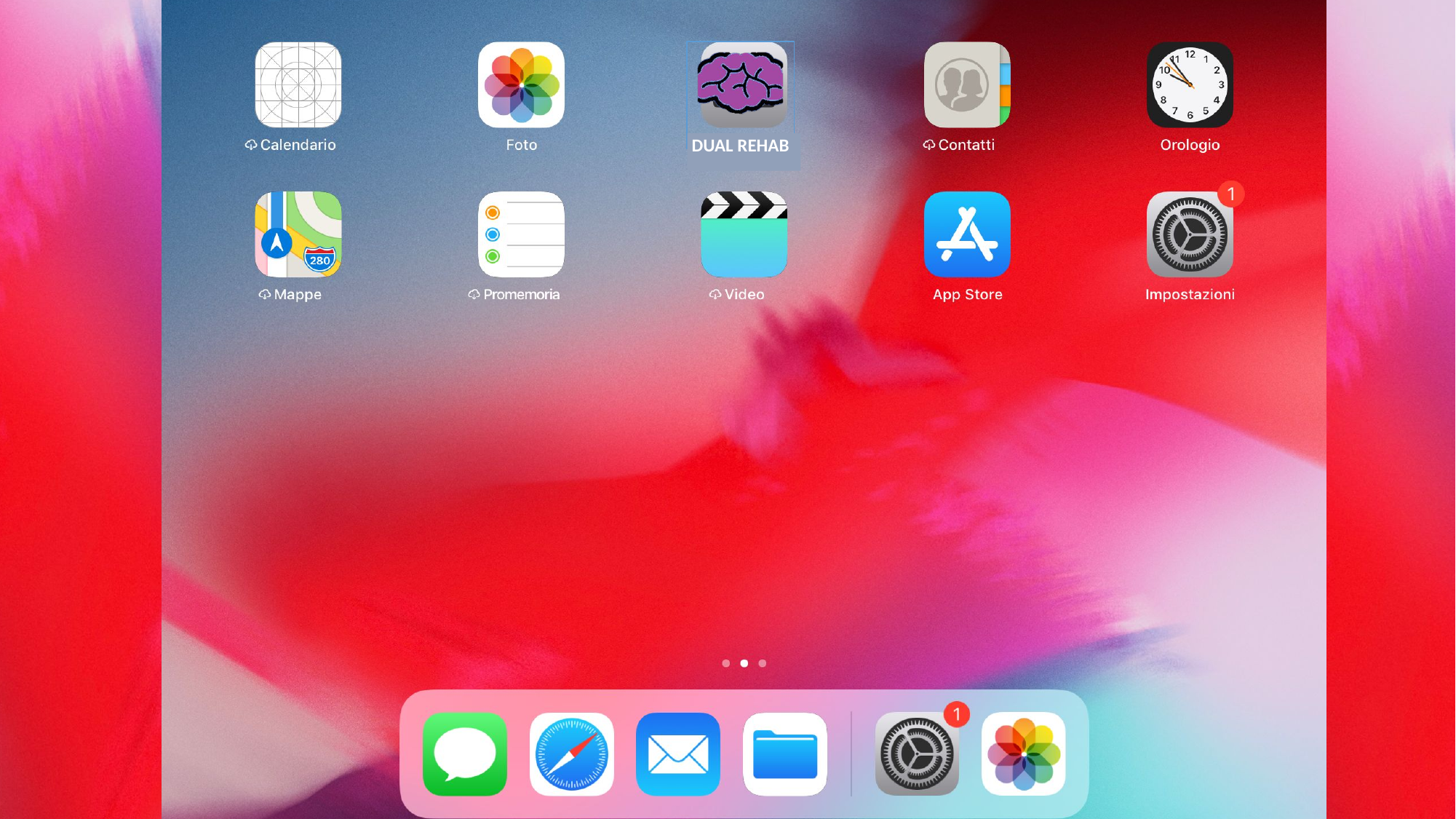

# DUAL REHAB
