# Supplementary figures and images for: 360°-Based Cognitive-Motor Training System for Older Adults With Cognitive Impairment: User-Centered Design and Evaluation Study
Source: JMIR Aging. 2026 Mar 13;9:e68032. doi: 10.2196/68032 (PMC13032090; doi:10.2196/68032)

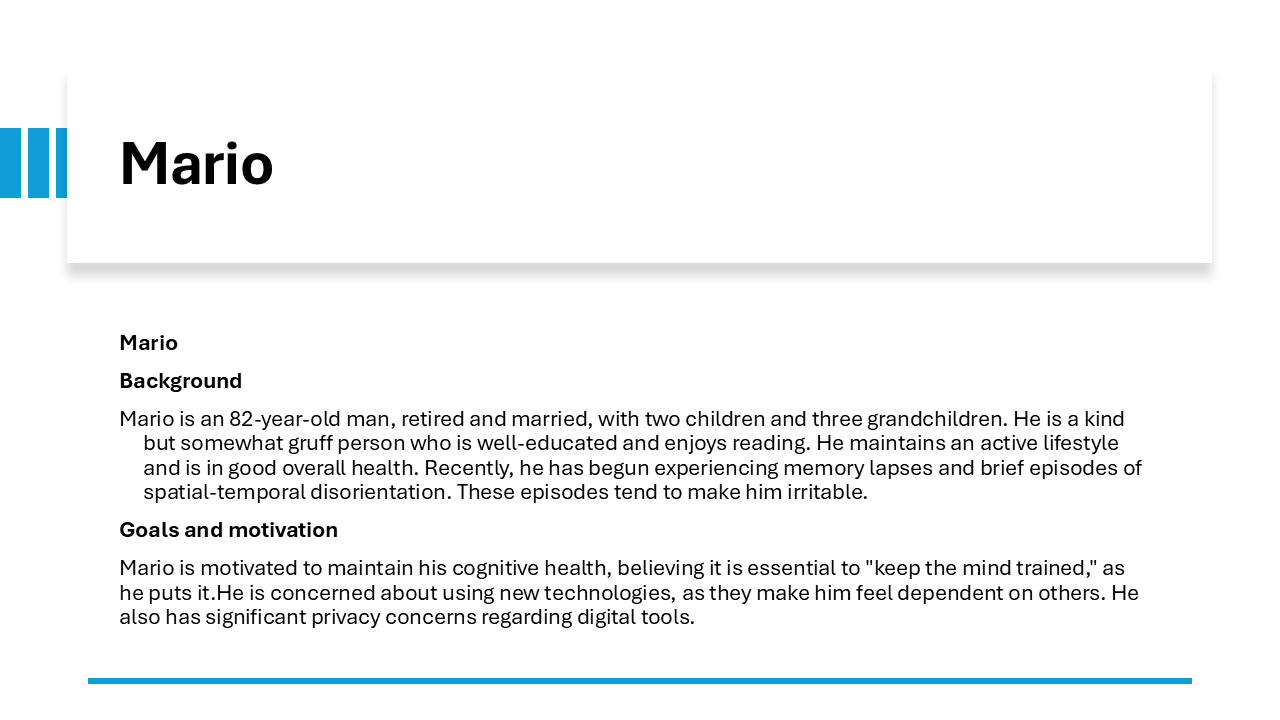

Supplement: Multimedia Appendix 2 [file aging_v9i1e68032_app2.png]

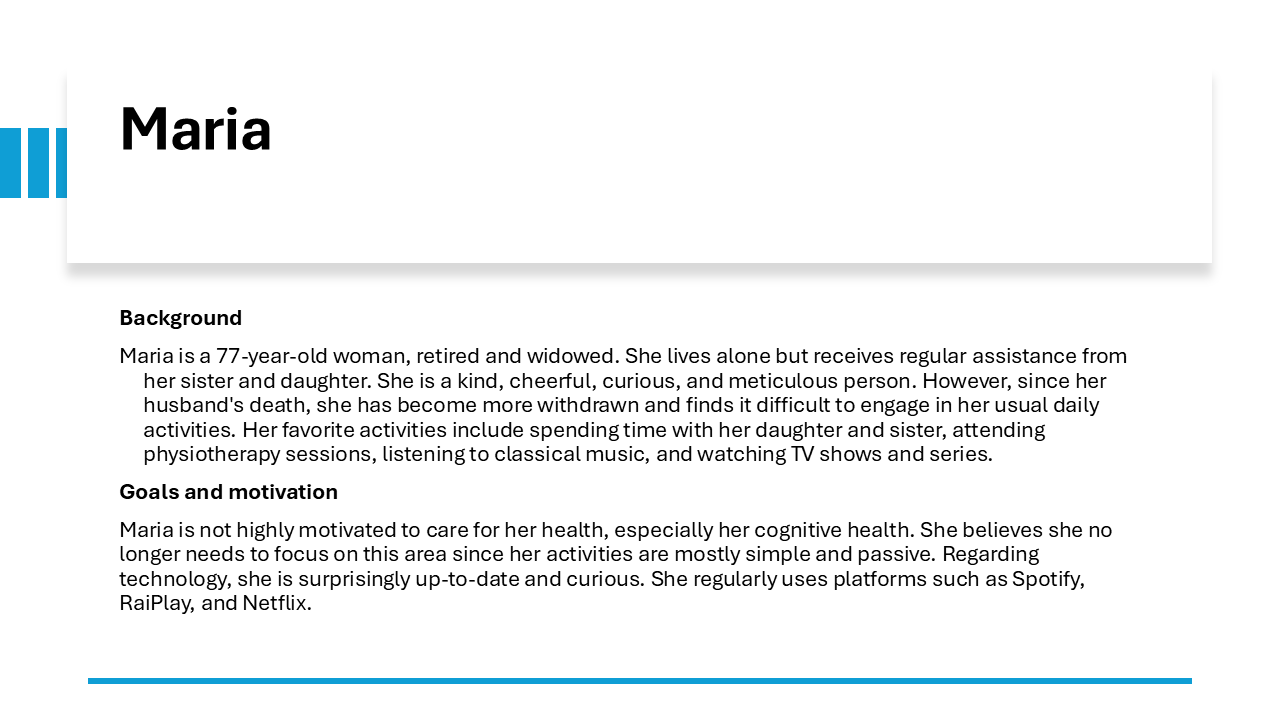

Supplement: Multimedia Appendix 3 [file aging_v9i1e68032_app3.png]
